# Mass cytometry reveals cladribine-induced resets among innate lymphoid cells in multiple sclerosis

**DOI:** 10.1038/s41598-022-24617-4

**Published:** 2022-11-27

**Authors:** F. T. Aglas-Leitner, P. Juillard, A. Juillard, S. N. Byrne, S. Hawke, G. E. Grau, F. Marsh-Wakefield

**Affiliations:** 1 Vascular Immunology Unit, School of Medical Sciences, Faculty of Medicine and Health, The University of Sydney, Sydney, Australia; 2 Medical University of Vienna, Spitalgasse 23, Vienna, Austria; 3 Centre for Immunology and Allergy Research, The Westmead Institute for Medical Research, Sydney, Australia; 4 Faculty of Medicine and Health, School of Medical Sciences, The University of Sydney, Sydney, Australia; 5 Central West Neurology and Neurosurgery, Orange, Australia; 6 Liver Injury & Cancer Group, Centenary Institute, Sydney, Australia; 7 Human Cancer & Viral Immunology Laboratory, The University of Sydney, Sydney, Australia; 8 Centenary Institute, Sydney, NSW Australia

**Keywords:** Autoimmunity, Innate lymphoid cells, Multiple sclerosis

## Abstract

Here we present a comprehensive mass cytometry analysis of peripheral innate lymphoid cell (ILC) subsets in relapsing/remitting MS (RRMS) patients prior to and after onset of cladribine tablets (CladT). ILC analysis was conducted on CyTOF data from peripheral blood mononuclear cells (PBMC) of MS patients before, 2 and 6 months after onset of CladT, and non-MS controls. Dimensionality reduction was used for immunophenotyping ILC subsets. CladT reduced all ILC subsets, except for CD56bright NK cells and ILC2. Furthermore, CD38+ NK cell and CCR6+ ILC3 were excluded from CladT-induced immune cell reductions. Post-CladT replenishment by immature ILC was noted by increased CD5+ ILC1 proportions at 2 months, and boosted CD38−CD56bright NK cell numbers at 6 months. CladT induce immune cell depletion among ILC but exclude CD56bright NK cells and ILC2 subsets, as well as CD38+ NK cell and CCR6+ ILC3 immunophenotypes. Post-CladT ILC expansions indicate ILC reconstitution towards a more tolerant immune system phenotype.

## Introduction

Multiple sclerosis (MS) is an autoimmune disease causing inflammatory demyelination in the central nervous system (CNS)^1^. Disease-modifying therapy (DMT) aims to curb MS disease activity by modulating the immune system^2^.

DMT with cladribine tablets (CladT) causes immune reconstitution by short-term, semi-selective immune cell depletion, resulting in the emergence of a new, expectedly more tolerant, immune system^2^. Once CladT are absorbed and intracellularly phosphorylated to cladribine triphosphate, cladribine triphosphate acts as a nucleoside analogue of deoxyadenosine, and thereby inhibits DNA synthesis and repair, resulting in consecutive cell apoptosis^3^.

Among innate lymphoid cells (ILC), the effects of CladT have thus far only been investigated on NK cells, which rapidly declined from baseline and demonstrated a nadir at week 5^4^. However, the impacts of CladT on other ILC subsets have not been studied.

ILC encompass five subtypes: NK cells, lymphoid tissue inducer cells, and three helper ILC subsets, namely ILC1, ILC2 and ILC3^5^. Together, ILC are characterized by their lymphoid morphology, but concomitant absence of adaptive antigen-specific receptors and myeloid/dendritic cell markers^6^. ILC constitute rapid innate immune reactions to viruses, tumors, extracellular microbes, and allergens^5,6^. Moreover, they are involved in tissue repair, tissue homeostasis and epithelial integrity^6,7^. Notwithstanding, imbalanced ILC enhance chronic inflammatory diseases^7^. ILC are therefore implicated in autoimmune diseases, such as type 1 diabetes mellitus, rheumatoid arthritis, systemic lupus erythematosus, systemic sclerosis, psoriasis, and spondylarthritis^8,9^.

To date, the role of ILC in MS is yet to be clarified in detail^9^. Increased pro-inflammatory ILC1 and/or ILC3 have been reported in peripheral blood of untreated MS patients^10,11^ as well as in CSF during early stages of the disease^12^. On the contrary, CD56bright NK cells have been proposed as immunoregulatory, and increase after various DMTs^13^.

Here we interrogate the impacts of CladT on NK cell and helper ILC subsets in MS patients by mass cytometry. CladT provoked marked reductions in the majority of circulating ILC subsets. However, certain ILC immunophenotypes were unaffected or even increased following CladT treatment, implying ILC-specific resets of the immune system towards more tolerogenicity, and thereby offering potential future therapeutic targets.

## Patients and methods

### Study participants

In this study relapsing–remitting MS (RRMS) patients were recruited from Neurology clinics at Central West Neurology and Neurosurgery, Orange, NSW and from the University of Sydney’s MS Clinic at the Brain and Mind Centre (BMC). MS was defined by McDonald 2017 criteria^14^. Age- and sex-matched blood donors without MS or other autoimmune disease (non-MS controls) were recruited from friends and relatives of MS patients, or other healthy volunteers. Patient data are shown in Supplementary Tables S1 and S2.

ILC derived from peripheral blood mononuclear cells (PBMC) were analyzed prior to and during the 6 months after the first dose of oral cladribine tablets (CladT), which comprised 5 consecutive days of CladT, followed by another consecutive 5 days 1 month later; a cumulative dose of 1.75 mg/kg was given. PBMC for mass cytometry analysis in this study were taken at baseline (*prior* to first dose of CladT), and at 2 (*2M*) and 6 months (*6M*) after their first intake of CladT (Supplementary Tables S2, S3). Untreated non-MS control subjects had blood taken at the same intervals as CladT-treated MS patients.

Furthermore, demographics (age, gender, date of first diagnosis), EDSS and MRI reviews were collected at various timepoints (Supplementary Table S3).

### Standard protocol approvals, registrations and patient consent

This study was performed according to the Declaration of Helsinki. Ethical consent for the study was obtained from the Research Integrity and Ethics Administration of the University of Sydney (2018/377). Written informed consent was obtained from all participants.

### Sample collection and preparation

Up to 50 mL of venous blood were collected in EDTA vacuette tubes (Greiner Bio-One International, Kremsmünster, Austria) from patients and controls. Blood was taken from MS patients prior to and 2, and 6 months after their first dose of CladT. Concurrently, the same numbers of blood tubes were also taken from age- and sex-matched non-MS control subjects at baseline, and then 2, and 6 months later. PBMC were then isolated from the blood using a Ficoll-Paque Plus (GE Healthcare, Chicago, Illinois, US) density separation gradient. Samples were cryopreserved in 5% DMSO/FBS for storage in liquid nitrogen prior to mass cytometry staining. Thawed cryopreserved Ficoll-isolated PBMCs were labelled with isotope-conjugated antibodies and examined by mass cytometry using the CyTOF2 system, Helios.

### Cell staining and analysis by mass cytometry

2.5 × 10^6^ cells were thawed at 37 °C and washed in RPMI medium^15^. Individual patient/timepoint samples were first barcoded with anti-human CD45 (on 4 different metal isotopes) and blocked with purified human FcR binding inhibitor (eBioscience Inc., San Diego, USA) for 30 min, such that four independent samples could be combined for further staining. To control for batch variability, PBMC taken from a single non-MS control (taken from a single timepoint) were included within each batch as an internal control, used across 22 batches. Samples were combined (7.5–10 × 10^6^ cells) and stained with cisplatin (Fluidigm, South San Francisco, CA, USA) for 5 min as a live/dead marker. Cells were stained with antibodies specific for the markers in Supplementary Table S4 for 30 min. These antibodies were purchased unlabeled in a carrier-protein-free and conjugated with the indicated metal isotope using the × 8 MaxPAR conjugation kit (Fluidigm) according to the manufacturer’s protocol. Conjugations done by the Ramaciotti Facility for Human Systems Biology are indicated in Supplementary Table S4. Cells were fixed in 4% paraformaldehyde (PFA) for 20 min prior to being incubated in Foxp3 permeabilisation buffer (eBioscience Inc.) for 15 min. Cells were then stained with anti-RORgt conjugated to AF647 for 45 min at room temperature (20–24 °C). After washing, cells were then stained with an intracellular antibody cocktail (indicated in Supplementary Table S4) for 45 min at room temperature. Cells were finally resuspended in DNA intercalator mix (500 nM iridium intercalator (Fluidigm) in 4% PFA) and left in the fridge until running on a CyTOF 2 Helios mass cytometer (Fluidigm) within 7 days. Cells were washed in Maxpar^®^ Cell Acquisition Solution (CAS) (Fluidigm) before being resuspended in 10% EQ four element beads (Fluidigm) in CAS at a concentration of 0.8 × 10^6^ cells mL^–1^.

### Analysis of CyTOF data

Data files (.fcs files), which had been acquired on the CyTOF 2 Helios mass cytometer (Fluidigm), were imported into FlowJo software (version v10.8.0, Becton Dickinson, Ashland, OR, USA). Samples were initially gated as shown in Supplementary Fig. S1 using FlowJo to identify CD45+ live single cells^15^. Of all acquired events, beads were first excluded with the use of 140Ce isotope. Cell aggregates were then removed by gating 191Ir DNA signal (from staining with an Ir-loaded DNA intercalator) versus event length. Live cells were identified based on cisplatin. CD45+ live PBMC were subsequently identified by barcoding with 4 different isotopes (104Pd, 106Pd, 108Pd, 110Pd).

**Figure 1:**
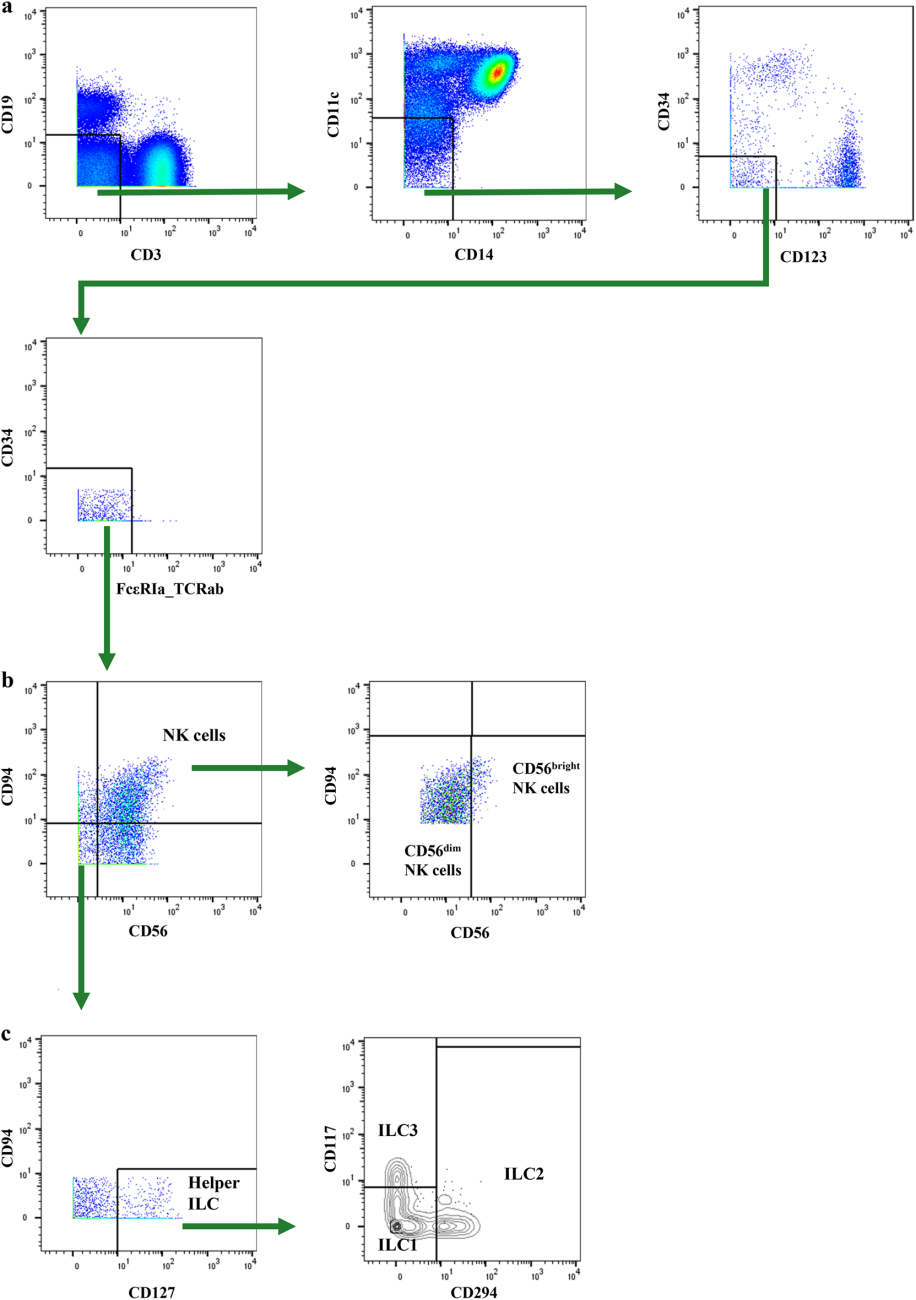
Gating strategy on CD45+ live PBMC to identify ILC subtypes. (**a**) Biaxial gating for Lin−(CD3−CD19−CD14−CD11c−CD123−CD34−FcεRIa−TCRab−) live CD45+ PBMC (peripheral blood mononuclear cells). (**b**) Natural killer (NK) cells were defined as Lin-CD56+CD94+ and subdivided into CD56dim and CD56bright NK cells. (**c**) Helper ILC were defined as Lin−CD56−CD94−CD127+ and subdivided into innate lymphoid cell (ILC) type 1 (CD294−CD117−), ILC2 (CD294+CD117+/−) and ILC3 (CD294−CD117+).

ILC were then defined as live CD45+ PBMC, negative for lineage markers (Lin−) CD3, CD19, CD14, CD11c, CD123, CD34, FcεRIa, and TCRαβ. Lin− cells were then sorted into Lin−CD56+CD94+ NK cells (and further subdivided into CD56bright and CD56dim NK cells), and Lin−CD56−CD94−CD127+ helper ILC (which were then subdivided into CD294-CD117− ILC1, CD294+CD117+/− ILC2 and CD294−CD117+ ILC3) (Fig. 1). Dimensionality reduction was done on all Lin−CD56−CD94−CD127+ helper ILC and Lin−CD56+CD94+ NK cells using ‘FIt-SNE’^16^, as part of the ‘Spectre’ package in R^15,17^. A subsample of up to 70,000 cells from all study participants (non-MS controls, MS patients from all timepoints and batch controls) were included for the generation of FIt-SNE plots. Dimensionality reduction plots were calculated using the following markers: CD1a, CD1c, CD5, CD21, CD23, CD38, CD56, CD80, CD86, CD94, CD103, CD117 (c-kit), CD120a, CD120b, CD161, CD184 (CXCR4), CD196 (CCR6), CD213α1, CD213α2, CD274 (PD-L1), CD294 (CRTH2), CD335 (NKp46), CD336 (NKp44), HLA-DR, GATA3, PAF-R (platelet-activating factor receptor), RORγt, T-bet (Supplementary Table S4). ILC immunophenotypes, which were identified with the help of FIt-SNE plots, were further analyzed in FlowJo software. To distinguish between positive versus negative surface marker expression of FIt-SNE-depicted ILC immunophenotypes, live CD45+ PBMC samples demonstrating low basal marker expression were considered as a negative control (Supplementary Fig. S2).

To calculate cell numbers, the percentage of the ILC as proportions of total PBMC were multiplied by PBMC counts of each study participant.

The median signal intensity of CD38 among total CD56+CD94+ and CD127 among Lin-CD56−CD94−CD127+ helper ILC was calculated and compared between groups.

### Statistical analysis

All statistics were calculated using packages available within R^18^, using Type III Sum of Squares for permuational multivariate analysis of variance (PERMANOVA)^15^.

To calculate differences between timepoints and disease status, a linear mixed-effects model was applied using the ‘lme4’ package^19^, in which the timepoints (*prior*, *2M*, *6M*) and disease status (MS or non-MS) were considered fixed effects, and patients were random effects. 4999 permutations were then run using the ‘permanova.lmer’ function as part of the ‘predictmeans’ R package to calculate p-values^20^. Permutational tests are powerful non-parametric tests, as they do not assume normal distribution or homogeneity of variance, and only assume data are exchangeable^15,21^. 4999 permutations were done to generate p-values. Holm’s corrections were used for multiple comparisons which were performed to compare various study timepoints (*prior* vs*. 2M, 2M* vs. *6M*, *prior* vs. *6M*, *non-MS prior* vs. *non-MS 2M*, *non-MS 2M* vs. *non-MS 6M*, *non-MS prior* vs. *non-MS 6M*, *prior* vs. *non-MS prior*, *2M* vs. *non-MS 2M*, *6M vs. non-MS 6M*).

A p-value < 0.05 was considered statistically significant. All plots were generated using GraphPad Prism software (GraphPad Software, San Diego, CA, USA) version 9.3.1 and 9.4.1.

## Results

To investigate the impacts of CladT on peripheral ILC in MS patients (Supplementary Tables S1, S2), we established a 43-parameter CyTOF panel (Supplementary Table S4), which allowed us to differentiate ILC subtypes in PBMC from MS patients at baseline (*prior*), as well as 2 (*2M*) and 6 months (*6M*) following the onset of CladT treatment (Supplementary Tables S2, S3). Furthermore, PBMC for ILC analysis were derived from *non-MS* controls (Supplementary Table S2) at baseline, as well as 2 and 6 months thereafter.

In total, 12 MS patients and 10 non-MS patients were analyzed in this study. No MS patients were on DMT at the time of blood sampling at study entry. MS patients prior to CladT treatment (prior, n = 12) were treatment-naïve (n = 4) or free from treatment for a minimum of 2 months prior to CladT (n = 8). All MS patients had low disability (EDSS range 0–2). MS activity was defined as new T2 and/or T1 Gadolinium-enhancing lesions in the 6 months before the onset of CladT (active MS: n = 9, inactive MS: n = 3).

ILC were defined as live CD45+ PBMC (Supplementary Fig. S1), negative for lineage markers (Lin−) CD3, CD19, CD14, CD11c, CD123, CD34, FcεRIa and TCRαβ (Fig. 1a). Lin− cells were gated into Lin−CD56+CD94+ NK cells (CD56bright and CD56dim NK cells) (Fig. 1b), and Lin−CD56−CD94−CD127+ helper ILC (CD294−CD117− ILC1, CD294+CD117+/− ILC2 and CD294−CD117+ ILC3) (Fig. 1c).

**Figure 2:**
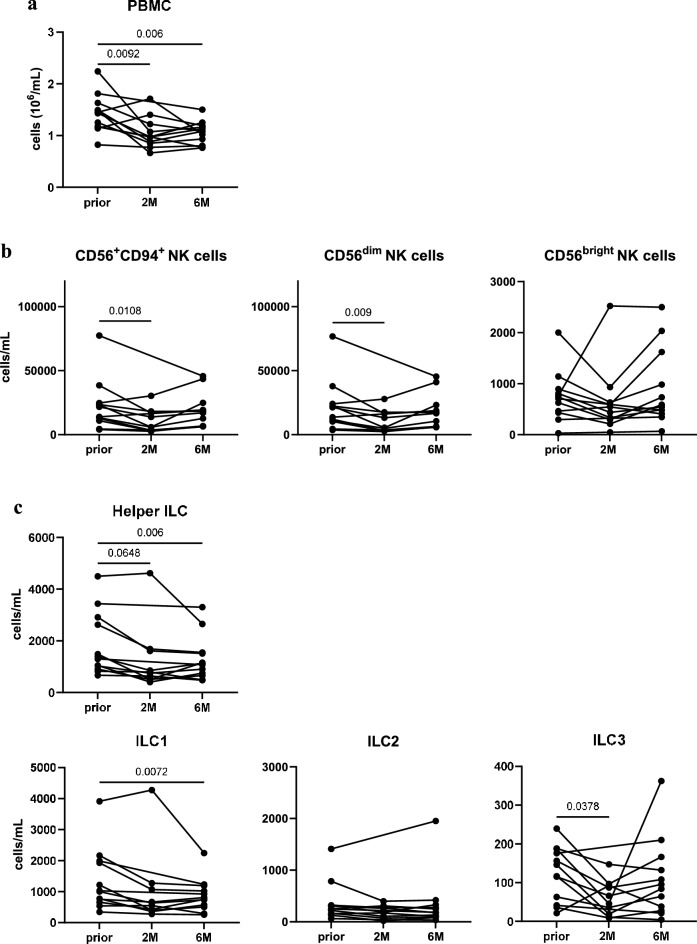
CladT reduce ILC but exempt CD56bright NK and ILC2 subsets. (**a**) Peripheral blood mononuclear cell (PBMC) count (10^6^/mL) across patients at baseline (*prior*, n = 12), and 2 (*2M*, n = 10) and 6 months (*6M*, n = 12) following the first administration of CladT. (**b**) Cell counts (cells/mL) of CD56+CD94+, CD56dim and CD56bright NK cells at *prior, 2M* and *6M*. NK cells were defined as live CD45+Lin−(CD3−CD19−CD14−CD11c−CD123−CD34−FcεRIa−TCRab−) CD56+CD94+, and further subdivided into CD56dim and CD56bright subpopulations. (**c**) Cell counts (cells/mL) of helper ILC (defined as live CD45+Lin−(CD3−CD19−CD14−CD11c−CD123−CD34−FcεRIa−TCRab−) CD56−CD94−CD127+helper ILC), and ILC1 (CD294−CD117−), ILC2 (CD294+CD117+/−) and ILC3 (CD294−CD117+) subsets at *prior* (n = 12), *2M* (n = 10) and *6M* (n = 12). A linear mixed-effects model was calculated to compare between MS patients before and after treatment. 4999 permutations were then run to calculate p-values. Holm’s corrections were used for multiple comparisons which were performed to compare the different timepoints (*prior*, *2M* & *6M*). p-values of p < 0.1 are indicated on the graphs.

Firstly, CladT treatment induced significant reductions in PBMC numbers at 2 (Fig. 2a) and 6 months post-administration (Fig. 2a). The decrease in PBMC was resembled by total and CD56dim NK cells at the *2M* (Fig. 2b) but not *6M* timepoint. At the same time, no marked alterations in CD56bright NK cells were apparent (Fig. 2b). Helper ILC and ILC1 reflected the significant decrease in PBMC at *6M* (Fig. 2c) but not *2M*. In contrast, ILC3 were diminished at the *2M* (Fig. 2c) but not *6M* timepoint, while ILC2 remained unaffected (Fig. 2c). Non-MS controls did not demonstrate any changes in PBMC or ILC throughout the study (Supplementary Fig. S3) and did not significantly differ from MS patients at any timepoint (Supplementary Fig. S4). Furthermore, the observed trends in helper ILC subset levels were not reflected by expression levels of CD127 on these cell types (measured as median signal intensity) (Supplementary Fig. S5).

To further elucidate the observed shifts in ILC upon CladT, ILC immunophenotypes were classified by FIt-SNE dimensionality reduction and assessed at all study timepoints.

**Figure 3:**
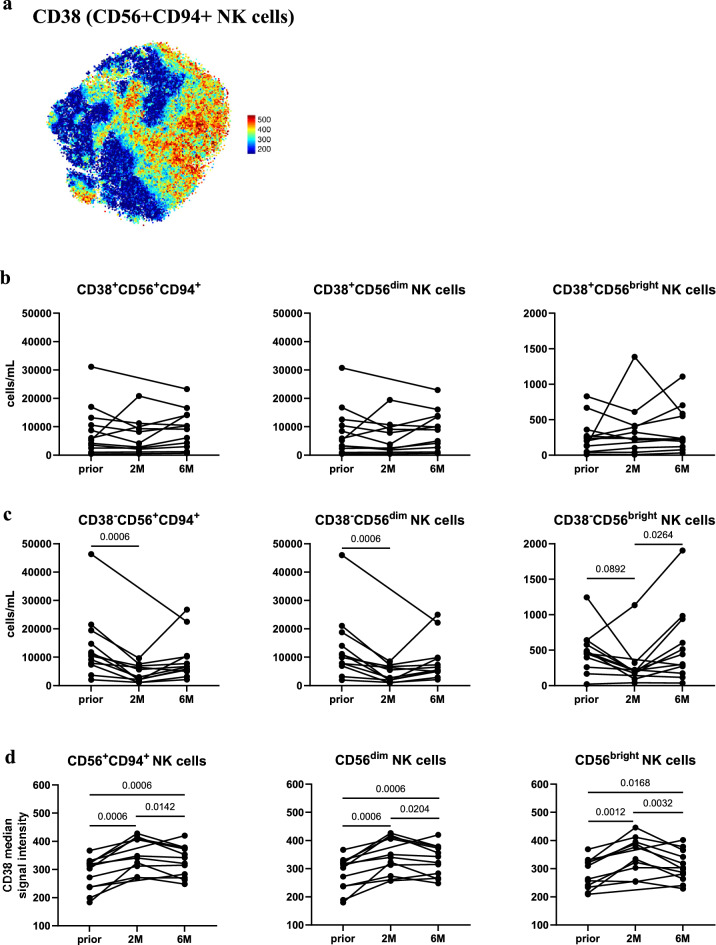
CD38+ but not CD38− NK cells remain unaffected by CladT. (**a**) Expression levels of CD38 among CD56+CD94+ NK cells on Fast Interpolation-based t-stochastic neighbor embedding (FIt-SNE) plots. A subsample of up to 70,000 cells from all study participants (non-MS controls, MS patients from all timepoints and batch controls) were included for the generation of FIt-SNE plots. (**b**) CD38+CD56+CD94+, CD38+CD56dim, CD38+CD56bright NK cells as cells/mL in MS patients at baseline (*prior*, n = 12), and 2 (*2M*, n = 10), and 6 months (*6M*, n = 12) following administration of CladT. (**c**) CD38-CD56+CD94+, CD38−CD56dim and CD38−CD56bright NK cells as cells/mL in MS patients *prior* (n = 12), *2M* (n = 10), and *6M* (n = 12) following administration of CladT. (**D**) CD38 median signal intensity of MS patients at *prior* (n = 12), *2M* (n = 10), and *6M* (n = 12) following administration of CladT. A linear mixed-effects model was calculated to compare between MS patients before and after treatment. 4999 permutations were then run to calculate p-values. Holm’s corrections were used for multiple comparisons which were performed to compare the different timepoints (*prior*, *2M* & *6M*). p-values of p < 0.1 are indicated on the graphs.

FIt-SNE of total NK cells revealed CD38+/− NK cell subpopulations (Fig. 3a). CD38 expression levels were not related to high or low expression of NK-cell defining markers CD56 or CD94 among total CD56+CD94+ NK cells (Supplementary Fig. S6), which was further reflected by similarly distributed CD38+ populations among NK cell subsets (34.90% of CD56+CD94+, 33.31% of CD56bright and 34.86% of CD56dim NK cells were CD38+ on average). Strikingly, CD38+NK cells were not affected by CladT (Fig. 3b). In contrast, CD38− total and CD38−CD56dim NK cells were significantly diminished at *2M* (Fig. 3c), reflecting the above-mentioned abatements in total and CD56dim NK cells upon CladT (Fig. 2b). Like CD56bright NK cells, CD38−CD56bright NK cells did not undergo any significant changes from *prior* to *2M* (Fig. 3c). However, they displayed an increment from *2M* to *6M* (Fig. 3c), which was not observed in total CD56bright NK cells (Fig. 2b). Furthermore, clear enhancements in CD38 median signal intensity among total CD56+CD94+, CD56bright and CD56dim NK cells were observed from *prior* to *2M* and *prior* to *6M*, with a slight decline from *2M* to *6M* (Fig. 3d), which were not apparent among non-MS participants (Supplementary Fig. S7).

**Figure 4:**
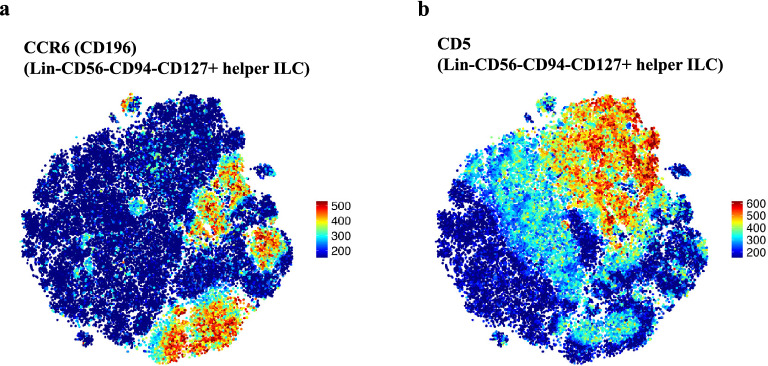
FIt-SNE depicts CCR6 (CD196) and CD5 expression on helper ILC. Expression levels of (**a**) CCR6 (CD196) and (**b**) CD5 among Lin−CD56−CD94−CD127+ helper ILC on Fast interpolation-based t-stochastic neighbor embedding (Fit-SNE) plots. A subsample of up to 70,000 cells from all study participants (non-MS controls, MS patients from all timepoints and batch controls) were included for the generation of FIt-SNE plots.

Next, FIt-SNE was performed on helper ILC to elucidate ILC immunophenotypes that are potentially involved in the observed ILC1 and ILC3 shifts upon CladT treatment. FIt-SNE differentiated helper ILC subsets ILC2 and ILC3 by their expression of CD294+ (ILC2) and CD117+ (ILC3), while ILC1 did not express these markers (Supplementary Fig. S8). FIt-SNE plots also identified small helper ILC populations of high HLA-DR, CD103, and CD335 expression, but did not demonstrate positive expression of co-stimulatory signals CD80 and CD86, or any expression of natural cytotoxicity receptor CD336 (Supplementary Fig. S9). However, FIt-SNE plots depicted clear bimodal CCR6 and CD5 expression by helper ILC immunophenotypes (Fig. 4a,b), which were further investigated.

**Figure 5:**
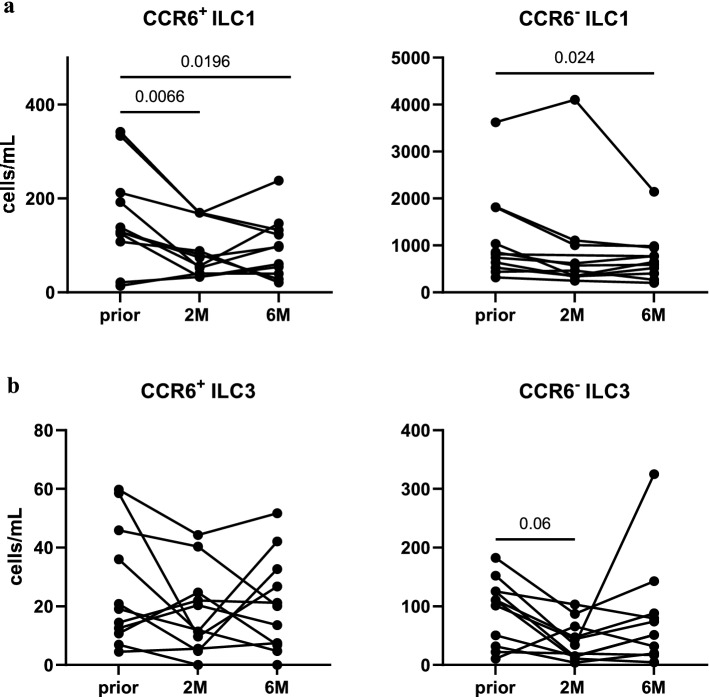
CCR6+ ILC3 are exempted from CladT-induced reductions in helper ILC. (**a**) CCR6+/− ILC1, and (**b**) CCR6+/− ILC3 as cells/mL in MS patients at baseline (*prior*, n = 11), and 2 (*2M*, n = 10), and 6 months (*6M*, n = 11) following administration of CladT. A linear mixed-effects model was calculated to compare between MS patients before and after treatment. 4999 permutations were then run to calculate p-values. Holm’s corrections were used for multiple comparisons which were performed to compare the different timepoints (*prior*, *2M* & *6M*). p-values of p < 0.1 are indicated on the graphs.

16.60% of total helper ILC were identified as CCR6+, of which 14.03% were ILC1, and 27.25% ILC3. Reductions in helper ILC1 and ILC3 upon CladT (Fig. 2c) were reflected by CCR6+ and CCR6- ILC1 (Fig. 5a), as well as CCR6- ILC3 (Fig. 5b). Strikingly, CCR6+ ILC3 were excluded from CladT-caused decreases in helper ILC (Fig. 5b). No significant shifts were observed among CCR6+/− helper ILC1 and ILC3 when analyzed as a proportion of total helper ILC (Supplementary Fig. S10).

**Figure 6:**
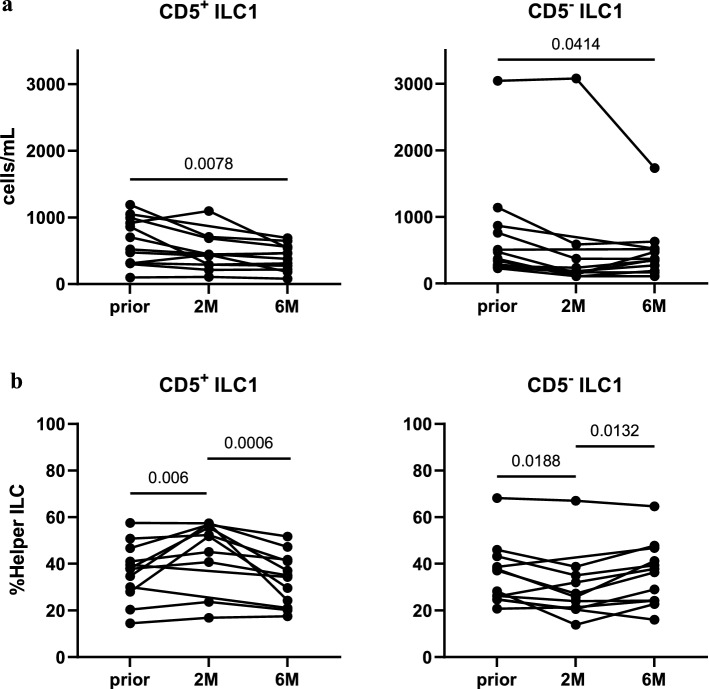
CD5+ ILC1 demonstrate short-term increments upon CladT within the helper ILC compartment. (**a**) CD5+ ILC1 and CD5− ILC1 as cells/mL in MS patients at baseline (*prior*, n = 12), and 2 (*2M*, n = 10), and 6 months (*6M*, n = 12) following administration of CladT. (**b**) CD5+ ILC1 and CD5−ILC1 as %Lin−CD56−CD94−CD127+ helper ILC (%Helper ILC) in MS patients at *prior* (n = 12), *2M* (n = 10), and *6M* (n = 12) following administration of CladT. A linear mixed-effects model was calculated to compare between MS patients before and after treatment. 4999 permutations were then run to calculate p-values. Holm’s corrections were used for multiple comparisons which were performed to compare the different timepoints (*prior*, *2M* & *6M*). p-values of p < 0.1 are indicated on the graphs.

Furthermore, 43.09% of helper ILC were identified as CD5+. Most CD5+ ILC were ILC1 (54.19% CD5+ ILC1), whereas merely 2.93% of ILC3 were CD5+. Similar to total ILC1 counts (Fig. 2c), CD5+ and CD5– ILC1 numbers exhibited marked declines at *6M* (Fig. 6a). Interestingly, when CD5+ ILC were analyzed as a percentage of helper ILC, they demonstrated a significant increase from baseline to *2M* (Fig. 6b), and a subsequent decline from *2M* to *6M* (Fig. 6b). In contrast, CD5− ILC1 significantly decreased from *prior* to *2M* (Fig. 6b), but recovered from *2M* to *6M* (Fig. 6b).

## Discussion

We present here a comprehensive mass cytometry analysis of circulating ILC subsets (CD56bright & CD56dim NK cells, ILC1, ILC2 & ILC3) in CladT-treated RRMS patients.

By semi-selective immune cell depletion, CladT is thought to induce the reconstitution of an immune system more tolerant of the CNS^2^. This likely occurs through the expansion of immune cells that remain despite immune reconstitution therapeutics, such as CladT^2^.

Among NK cells, CladT spared CD56bright NK cells, which are known for autologous CD4+ T cell suppression^22^. This function is impaired in MS patients^22^ but is reversed by several DMTs through numeral, proportional and/or functional enhancements in CD56bright NK cells^13,22^. Similarly, Moser et al.^23^ reported CD56bright regulatory NK cells with non-significant numeral expansions upon CladT. In contrast, enhanced circulating CD56dim NK cell numbers^24^ and proportions^24,25^ are associated with MS disease activity^24,25^, while therapy-naïve MS patients with stable disease course demonstrate reduced CD56dim NK cells compared to healthy subjects^26^. Therefore, CladT-induced reductions of CD56dim NK cells but unaltered CD56bright NK cell levels imply an MS-beneficial immune reconstitution within the NK cell compartment.

Furthermore, we found that CD38+ NK cells were resistant to CladT-induced immune cell depletion and demonstrated enhanced CD38 expression upon CladT. CD38+ NK cells may impose regulatory functions in MS, as CD38+CD56bright NK cells are implicated in adenosine production, which consequently inhibits autologous CD4+ T cell proliferation^22^. Interestingly, the anti-CD25 monoclonal antibody daclizumab increases CD38 expression by CD56bright NK cells^27^. Moreover, CD38 characterizes mature NK cells, which demonstrate efficacious cytokine production and cytolytic functions^28^. We also noted expansions of CD38−CD56bright from *2M* to *6M*. Thus, immune cell depletion by CladT may foster subsequent expansion of immature CD38−CD56bright NK cells, which, once matured to CD38+ NK cells, have MS-beneficial effects.

Similarly, CladT caused selective depletion of helper ILC subsets. Only ILC1 and ILC3, but not ILC2, were significantly reduced upon CladT. Coherently, observations in MS patients and/or experimental autoimmune encephalomyelitis (EAE) models have pointed towards pathogenic influences of ILC1 and ILC3^10–12,29^, but beneficial impacts of ILC2^29^.

In addition, we delineated CCR6 and CD5+ helper ILC immunophenotypes by FIt-SNE, which are of particular interest in CladT-treated MS patients.

Firstly, CCR6+ ILC3 infiltrate the CNS, where they stimulate myelin-specific T cells in EAE^30^. Concomitantly, cladribine impedes lymphocyte transition across the blood brain barrier^31,32^. Hence, our observations in which CCR6+ ILC3 were excluded from CladT-induced peripheral immune cell reduction suggest reduced migration of CCR6+ ILC3 to the CNS following treatment with CladT.

Secondly, our reported increases in CD5+ ILC1 proportions among helper ILC imply short-term replenishment of helper ILC upon CladT-induced immune cell depletion, as CD5+ ILC encompass immature ILC which however downregulate expression of CD5 once they acquire effector functions^33^. Hence, proportional expansions in CD5+ ILC1 suggest that CladT reduces mature ILC while exempting their immature precursors.

Limitations to our study include a relatively low *n*-number of study participants, which did not enable separate analysis of active versus non-active MS subjects. Furthermore, the low occurrence of ILC in circulating blood was another challenge in our analyses. No MS patients relapsed during the observational period. Hence, the effect on the ILC compartment during MS relapse needs to be addressed in future studies.

Moreover, ratios of deoxycytidine kinase (DCK) and 5′-nucleotidase (5′-NT), which activate and inactivate CladT metabolites respectively, are presumed as the determinant of its cell type-specificity^3,4^. To our knowledge, DCK:5′-NT ratios have so far only been measured among NK cells^4,34^ but not helper ILC. Strikingly, CD56bright and CD56dim NK cells show comparable DCK:5′-NT ratios^4,34^, but were affected differently by CladT in our study. Possibly, CladT-induced effects on other immune cells influence ILC subtypes indirectly, especially as ILC display essential crosstalk to other immune cells^5^. Lastly, future studies should evaluate DCK enzyme activity^34^, and ADA enzyme levels among ILC^35^, which may also influence vulnerability to CladT^34,35^.

In summary, CladT caused dramatic reductions in the majority of ILC, but spared potentially MS-inhibitory CD56bright NK cells, ILC2, and CD38+ NK cells. Consistent peripheral levels of CCR6+ ILC3 may further depict CladT-altered migration of pro-inflammatory immune cells to the CNS. Replenishment of the ILC compartment upon CladT is expected, as enhancements in CD38−CD56bright NK cells and CD5+ ILC1 are likely to comprise of immature ILC. Hence, we suggest ILC-specific resets of the immune system towards more tolerogenicity, with influential implications for the development of future therapeutic targets.

## Supplementary Information

Supplementary Information.

## Data Availability

The datasets used and analyzed during the current study are available from the corresponding author on reasonable request.

## Abbreviations

*2M*: 2 months after cladribine tablets
5′-NT: 5′-Nucleotidase
*6M*: 6 months after cladribine tablets
BMC: Brain and Mind Centre
CAS: Cell Acquisition Solution
CNS: Central nervous system
CladT: Cladribine tablets
DCK: Deoxycytidine kinase
DMT: Disease-modifying therapy
EAE: Experimental autoimmune encephalomyelitis
ILC: Innate lymphoid cell
MS: Multiple sclerosis
PFA: Paraformaldehyde
PBMC: Peripheral blood mononuclear cells
PERMANOVA: Permuational multivariate analysis of variance
RRMS: Relapsing/remitting MS
