# Supplementary material for: Mass cytometry reveals cladribine-induced resets among innate lymphoid cells in multiple sclerosis
Source: Sci Rep. 2022 Nov 27;12:20411. doi: 10.1038/s41598-022-24617-4 (PMC9701791; doi:10.1038/s41598-022-24617-4)
Supplement: Supplementary file 1 — Supplementary Information. [file 41598_2022_24617_MOESM1_ESM.pdf]

Supplementary Material:

Mass cytometry reveals cladribine-induced resets among innate lymphoid cells in multiple sclerosis

Aglas-Leitner FT[1,2], Juillard P[1], Juillard A[1], Byrne, SN[3,4], Hawke S[1,5], Grau GE[1,*], Marsh-Wakefield F[1,6,7,*]

[1]Vascular Immunology Unit, School of Medical Sciences, Faculty of Medicine and Health, The University of Sydney, Sydney, Australia

[2]Medical University of Vienna, Spitalgasse 23, Vienna, Austria

[3]Centre for Immunology and Allergy Research, The Westmead Institute for Medical Research, Sydney, Australia

[4]Faculty of Medicine and Health, School of Medical Sciences, The University of Sydney, Sydney, Australia

[5]Central West Neurology and Neurosurgery, Orange, Australia

[6]Liver Injury & Cancer Group, Centenary Institute, Sydney, Australia

[7]Human Cancer & Viral Immunology Laboratory, The University of Sydney, Sydney, Australia.

[*]Corresponding authors contributed equally.

Keywords:

Cladribine tablets, innate lymphoid cells, relapse-remitting multiple sclerosis, mass cytometry

Correspondence:

Dr. Felix Marsh-Wakefield

Centenary Institute, NSW, Australia

T: +61 2 8627 1746

f.marsh-wakefield@centenary.org.au

felix.marsh-wakefield@sydney.edu.au

Supplementary Table S1: Study participant age and sex (female (F) versus male (M)).

PATIENT	SEX	AGE at *"prior"* study timepoint	AGE at first diagnosis
MS patients	*83% F*	*Median = 37.9*	*Median = 33.8*
**MS01**	F	25.2	24.8
**MS02**	F	37.2	20.0
**MS03**	F	40.2	35.0
**MS04**	M	36.7	36.3
**MS05**	F	35.8	29.5
**MS06**	F	37.4	35.0
**MS07**	F	44.8	44.7
**MS08**	F	45.5	27.0
**MS09**	F	38.4	39.8
**MS10**	F	38.5	33.0
**MS11**	M	34.7	30.3
**MS12**	F	49.9	34.5
Non-MS patients	*80% F*	*Median = 39.7*	
**Non-MS01**	F	27.5	
**Non-MS02**	F	40.8	
**Non-MS03**	F	36.8	
**Non-MS04**	M	39.0	
**Non-MS05**	M	42.3	
**Non-MS06**	F	41.4	
**Non-MS07**	F	34.1	
**Non-MS08**	F	54.9	
**Non-MS09**	F	40.3	
**Non-MS10**	F	23.5	

Supplementary Table S2: Treatment regimen and disease activity of MS participants

PATIENT	MONTHS SINCE FIRST COURSE OF CLADRIBINE				PREVIOUS DMT	MONTHS SINCE LAST TREATMENT PRIOR TO CLADRIBINE	ACTIVE MS*	EDSS SCORE
	Prior	*2M*	*6M*	*Relapse*				
**MS PATIENTS** *n=12*	*n=12*	*n=10*	*n=12*	*n=0*	*previous DMT: n=8 Treatment-naïve: n=4*	*Median = 2 Range = 2-15*	*Active: n=9 Non-active: n=3*	*Median = 0 Range = 0-2*
**MS01****	X		X		Dimethyl fumarate	2	Yes	0
**MS02**	X	X	X		—/—	—/—	Yes	0
**MS03**	X	X	X		Fingolimod	15	Yes	0
**MS04**	X	X	X		—/—	—/—	Yes	1
**MS05**	X	X	X		—/—	—/—	Yes	0
**MS06**	X	X	X		Fingolimod	2	No	0
**MS07**	X	X	X		—/—	—/—	Yes	0
**MS08**	X	X	X		Fingolimod	15	No	0
**MS09**	X	X	X		Fingolimod	3	Yes	2
**MS10**	X	X	X		Fingolimod	2	Yes	0
**MS11**	X		X		Fingolimod	2	Yes	0
**MS12**	X	X	X		Fingolimod	2	No	0
**Non-MS01**	X		X					
**Non-MS02**	X	X	X					
**Non-MS03**	X							
**Non-MS04**	X	X						
**Non-MS05**	X	X						
**Non-MS06**	X	X	X					
**Non-MS07**	X							
**Non-MS08**	X		X (9M***)					
**Non-MS09**	X		X					
**Non-MS10**	X	X	X					

*Defined as new T2 and/or T1 Gad-enhancing lesions in the 6 months prior to starting cladribine

**no CD196 antibody staining of PBMC from this patient

***Sample taken 9 months after initial sample.

Supplementary Table S3: Study timeline

ASSESSMENT	Baseline	Month 2	Month 6
Demographics (age, gender, date of first diagnosis)	x		
Cladribine therapy	x		
EDSS	x		x
Review of MRI**	x		x
Blood sampling	x	x	x
CyTOF analysis of PBMC	x	x	x

**MRI performed as per treating neurologist.

Supplementary Table S4: CyTOF antibody panel

ANTIBODY	ISOTOPE	CLONE	COMPANY	FIT-SNE PLOTS
**CD1a**	167Er*	HI149	BioLegend	✓
**CD1c**	163Dy*	L161	BioLegend	✓
**CD3**	115In	UCHT1	BioLegend	
**CD4**	168Er*	SK3	BioLegend	
**5-HT2A**	168Er*	9B11.1	Merck	
**CD5**	148Nd	UCHT2	BioLegend	✓
**CD8a**	89Y*	RPA-T8	BD Biosciences	
**CD11c**	172Yb	Bu15	BioLegend	
**CD14**	160Gd*	M5E2	BD Biosciences	
**CD19**	142Nd*	HIB19	BioLegend	
**CD21**	152Sm*	BU32	BioLegend	✓
**CD23**	169Tm*	EBVCS-5	BioLegend	✓
**CD34**	166Er*	581	BD Biosciences	
**CD38**	145Nd	HIT2	BioLegend	✓
**CD45†**	104Pd*	HI30	BioLegend	
**CD45†**	106Pd*	HI30	BioLegend	
**CD45†**	108Pd*	HI30	BioLegend	
**CD45†**	110Pd*	HI30	BioLegend	
**CD56**	113In*	NCAM16.2	BD Biosciences	✓
**CD80**	162Er*	L307.4	BD Biosciences	✓
**CD86**	156Gd*	IT2.2	BD Biosciences	✓
**CD94**	141Pr*	DX22	BioLegend	✓§
**CD103**	155Gd	Ber-ACT8	BioLegend	✓
**CD117 (c-Kit)**	143Nd*	104D2	BioLegend	✓
**CD120a**	158Gd	REA252	Miltenyi Biotec	✓
**CD120b**	159Tb	3G7A02	BioLegend	✓
**CD123**	151Eu*	6H6	BioLegend	
**CD127**	165Ho*	A019D5	BioLegend	
**CD161 (KLRB1)**	164Er*	DX12	BD Biosciences	✓

**CD184 (CXCR4)**	175Lu*	12G5	BD Biosciences	✓
**CD196 (CCR6)**	154Sm*	REA190	Miltenyi Biotec	✓
**CD213a1**	170Er	SS12B	BioLegend	✓
**CD213a2**	176Yb	REA308	Miltenyi Biotec	✓
**CD274 (PD-L1)**	161Dy*	29E.2A3	BioLegend	✓
**CD294 (CRTH2)**	153Eu	BM16	BioLegend	✓
**CD335 (NKP46)**	171Yb*	9E2 NKp46	BioLegend	✓
**CD336 (NKP44)**	149Sm	P44-8	BioLegend	✓
**FCeR1a**	146Nd*	AER-37 (CRA-1) T10B9.1A-31	BioLegend	
**TCRab**				
**GATA3 ‡**	139La	TWAJ	eBioscience	✓
**HLA-DR**	174Yb*	L243	BioLegend	✓
**PAF-R (platelet-activating factor receptor)**	150Nd	11A4, Clone 21	Cayman Chemicals	✓
**RORgt ‡**	147Sm*	Primary antibody: RORgt-AF647 conjugate, clone Q21-559.	RORgt from BD Biosciences; anti-Cy5 from Sigma	✓
		Secondary antibody: anti-Cy5, clone CY5-15.		
**T-bet ‡**	209Bi*	4B10	BD Biosciences	✓
**TCRgd**	144Nd*	B1	BioLegend	
**TCR Va 7.2**	173Yb*	3C10	BioLegend	

*Isotopes conjugated by the Ramaciotti Facility for Human Systems Biology, The University of Sydney, Australia.

†CD45 was used for barcoding to allow up to three samples (plus internal control) to be stained together.

§CD94 was only selected for dimensionality reduction plots for Lin-CD56-CD94-CD127+ helper ILC but not Lin-CD56+CD94+ NK cells.

‡Markers stained intracellularly.

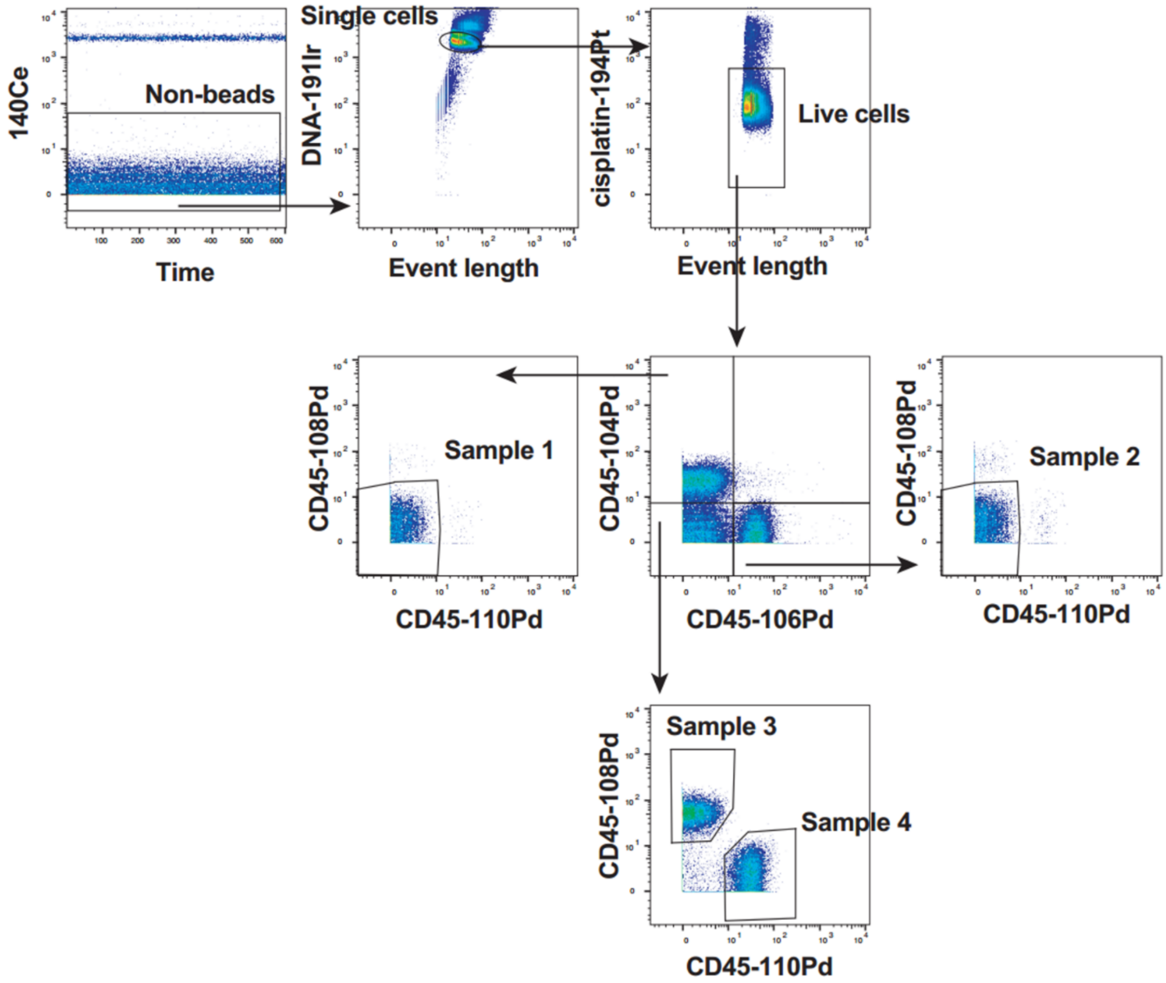

Supplementary Figure S1: Gating strategy for CD45+ PBMC

Of all acquired events, beads were first excluded with the use of 140Ce isotope. Cell aggregates were then removed by gating 191Ir DNA signal (from staining with an Ir-loaded DNA intercalator) versus event length. Live cells were identified based on cisplatin. Live CD45+ PBMC were then identified by barcoding with 4 different isotopes (104Pd, 106Pd, 108Pd, 110Pd).

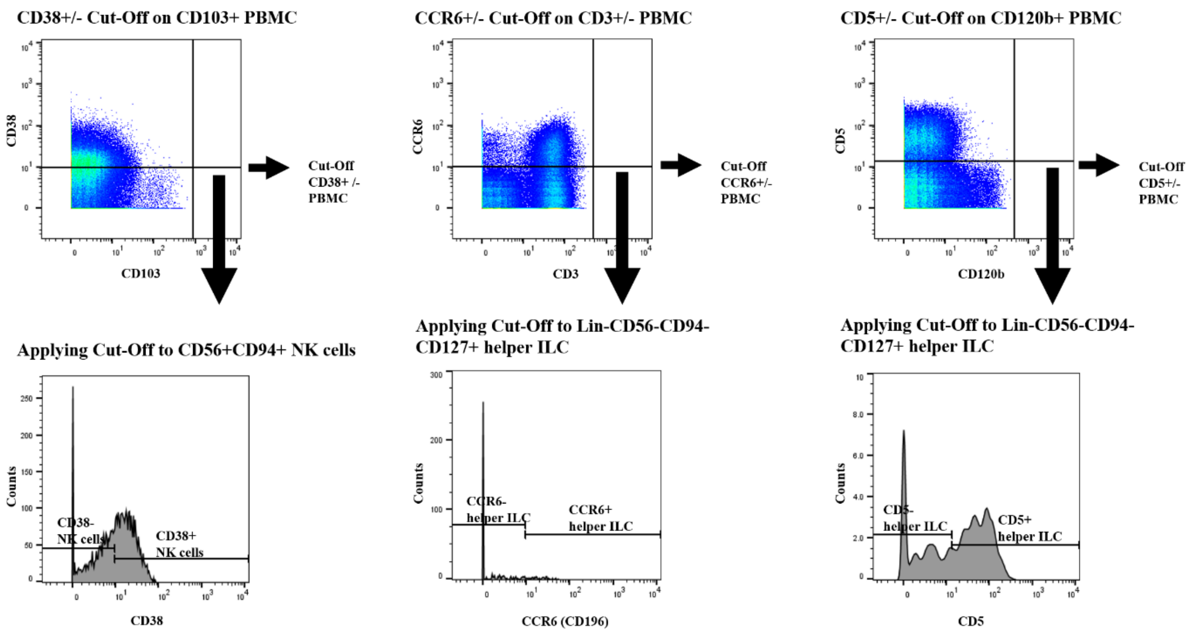

Supplementary Figure S2: Identification of ILC immunophenotypes

Due to their low expression of CD38, live CD45+CD103+ PBMC were considered as exemplary CD38- population. The thereby determined cut-off was then employed to differentiate between CD38+/- CD56+CD94+, CD56dim and CD56bright NK cells. CCR6 expression among live CD45+CD3+ and CD3- PBMC allowed the identification of CCR6+ and CCR6- PBMC populations. This cut-off was then used to identify CCR6+ and CCR6- Lin-CD56-CD94-CD127+ helper ILC, ILC1 & ILC3. Live CD45+ CD120b+ PBMC exhibited low CD5 expression and were hence used as a cut-off to distinguish CD5+ from CD5- Lin-CD56-CD94-CD127+ helper ILC & ILC1.

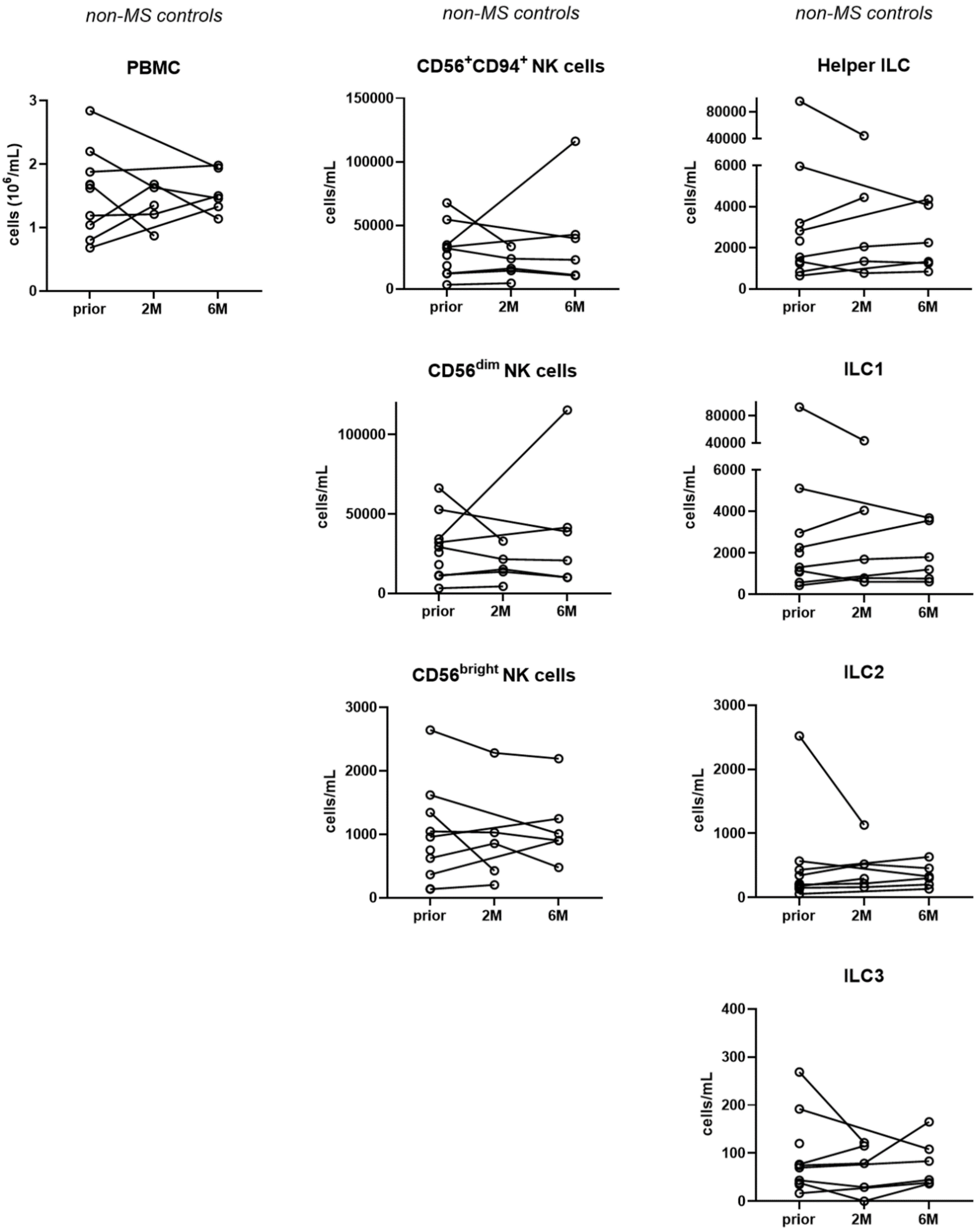
non-MS controls
non-MS controls
non-MS controls
PBMC
cells (10^6/mL)
prior
2M
6M
CD56+CD94+ NK cells
cells/mL
Helper ILC
CD56dim NK cells
ILC1
CD56bright NK cells
ILC2
ILC3

Supplementary Figure S3: No alterations in PBMC or ILC among non-MS control participants.

Peripheral blood mononuclear cell (PBMC) count (10^6/mL) across non-MS control participants at baseline (*prior*, n = 10), and two (*2M*, n = 5) and six months (*6M*, n = 6) thereafter. Cell counts (cells/mL) of CD56+CD94+, CD56dim and CD56bright NK cells at *prior, 2M* and *6M*. NK cells were defined as CD45+Lin- (CD3-CD19-CD14-CD11c-CD123-CD34-FcεRIa-TCRab-) CD56+CD94+, and further divided into CD56dim and CD56bright subpopulations. Cell counts (cells/mL) of helper ILC (defined as live CD45+ Lin- (CD3-CD19-CD14-CD11c-CD123-CD34-FcεRIa-TCRab-) CD56-CD94-CD127+ helper ILC), and ILC1 (CD294-CD117-), ILC2 (CD294+CD117+/-) and ILC3 (CD294-CD117+) subsets at *prior*, *2M* and *6M*. A linear mixed-effects model was calculated for comparison of different study timepoints. 4,999 permutations were then run to calculate p-values. Holm's corrections were used for multiple comparisons which were performed to compare the different timepoints (*prior*, *2M* & *6M*). P-values of $p<0.1$ are indicated on the graphs.

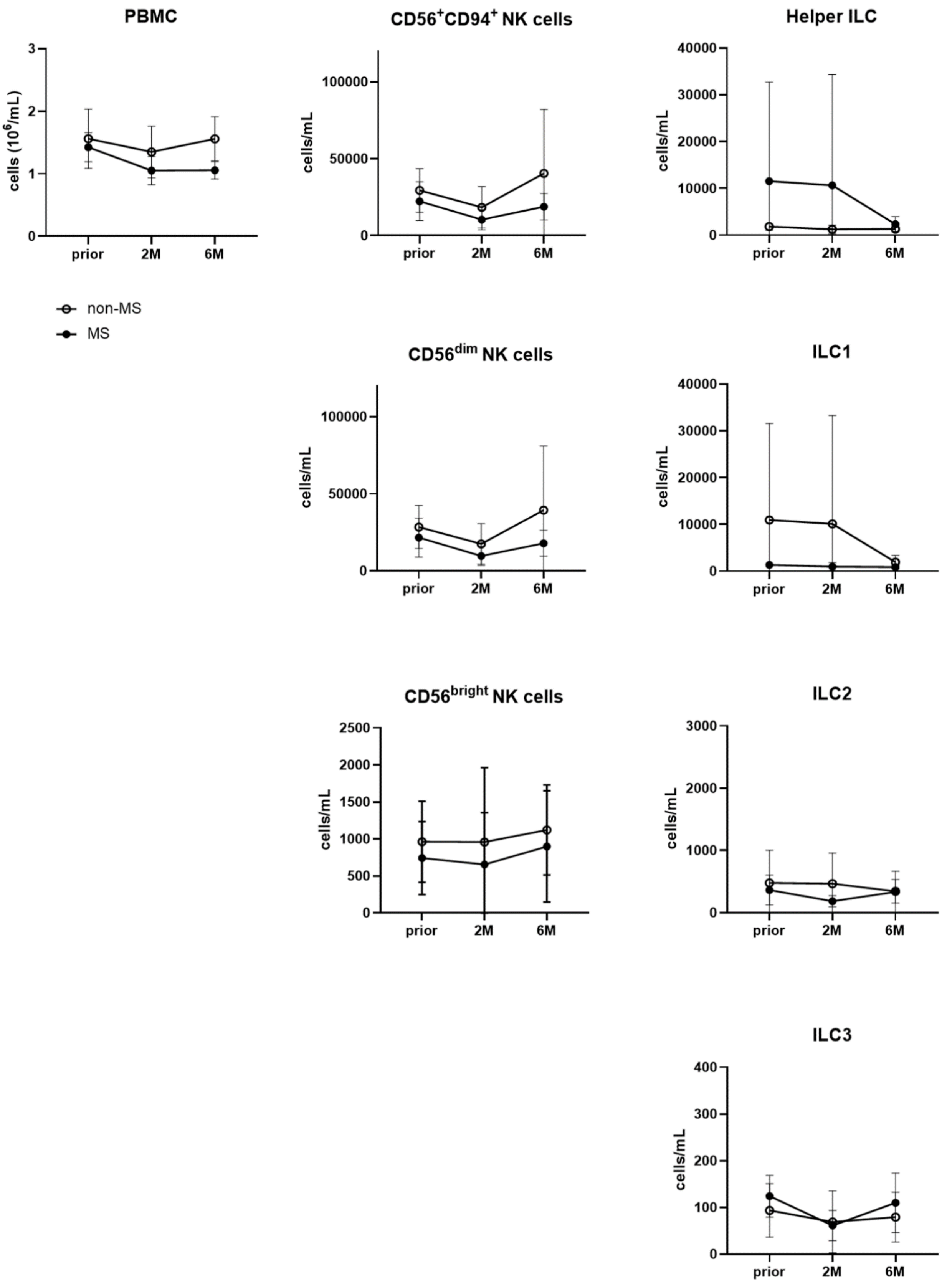
PBMC
cells (10^6/mL)
0
1
2
3
prior
2M
6M
non-MS
MS
CD56⁺CD94⁺ NK cells
cells/mL
0
50000
100000
Helper ILC
0
10000
20000
30000
40000
CD56dim NK cells
ILC1
CD56bright NK cells
0
500
1000
1500
2000
2500
ILC2
1000
2000
3000
ILC3
100
200
300
400

Supplementary Figure S4: PBMC and ILC levels do not differ between non-MS control subjects and cladribine-treated MS patients.

PBMC were measured as cells (10^6/mL), and CD56+CD94+, CD56dim, CD56bright NK cells, as well as Lin-CD56-CD94-CD127+ helper ILC, ILC1, ILC2 and ILC3 as cells/mL in *non-MS* control subjects (*prior* (n = 10), *2M* (n = 5), and *6M* (n = 6)) and cladribine-treated patients at baseline (*prior*, n = 12), and subsequently two months (*2M*, n = 10) and six months (*6M*, n = 12) later. A linear mixed-effects model was calculated between groups, with a permutations test to calculate p-values. Holm's corrections were used for multiple comparisons to compare individual patients. Three multiple comparisons were performed: *prior non-MS* versus *prior MS*, *2M non-MS* versus *2M MS*, and *6M non*-MS versus *6M MS*. Means and 95% confidence intervals are demonstrated by horizontal lines. No p-values of $p<0.1$.

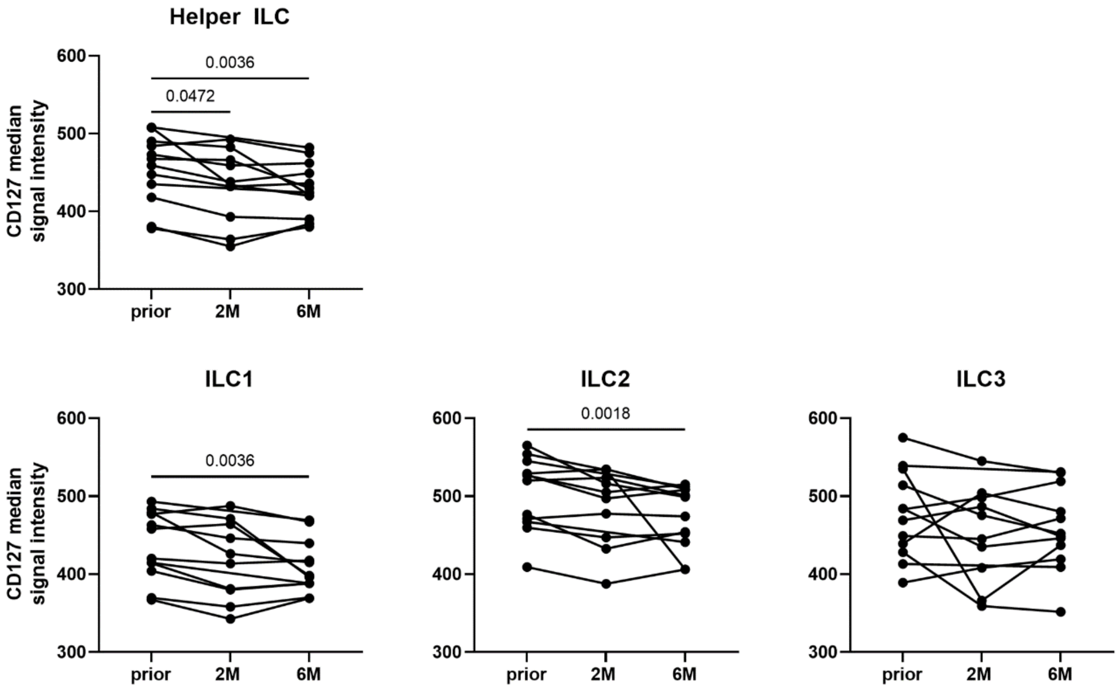

Supplementary Figure S5: CD127 median signal intensity among Lin-CD56-CD94-CD127+ helper ILC subsets before and after intake of cladribine in MS patients.

Median signal intensity of CD127 among Lin-CD56-CD94-CD127+ helper ILC, ILC1, ILC2 and ILC3 was calculated at *prior* (n = 12), *2M* (n = 10) and *6M* (n = 12) following intake of cladribine in MS patients. A linear mixed-effects model was calculated to compare between MS patients before and after treatment. 4,999 permutations were then run to calculate p-values. Holm's corrections were used for multiple comparisons which were performed to compare the different timepoints (*prior*, *2M* & *6M*). P-values of p<0.1 are indicated on the graphs.

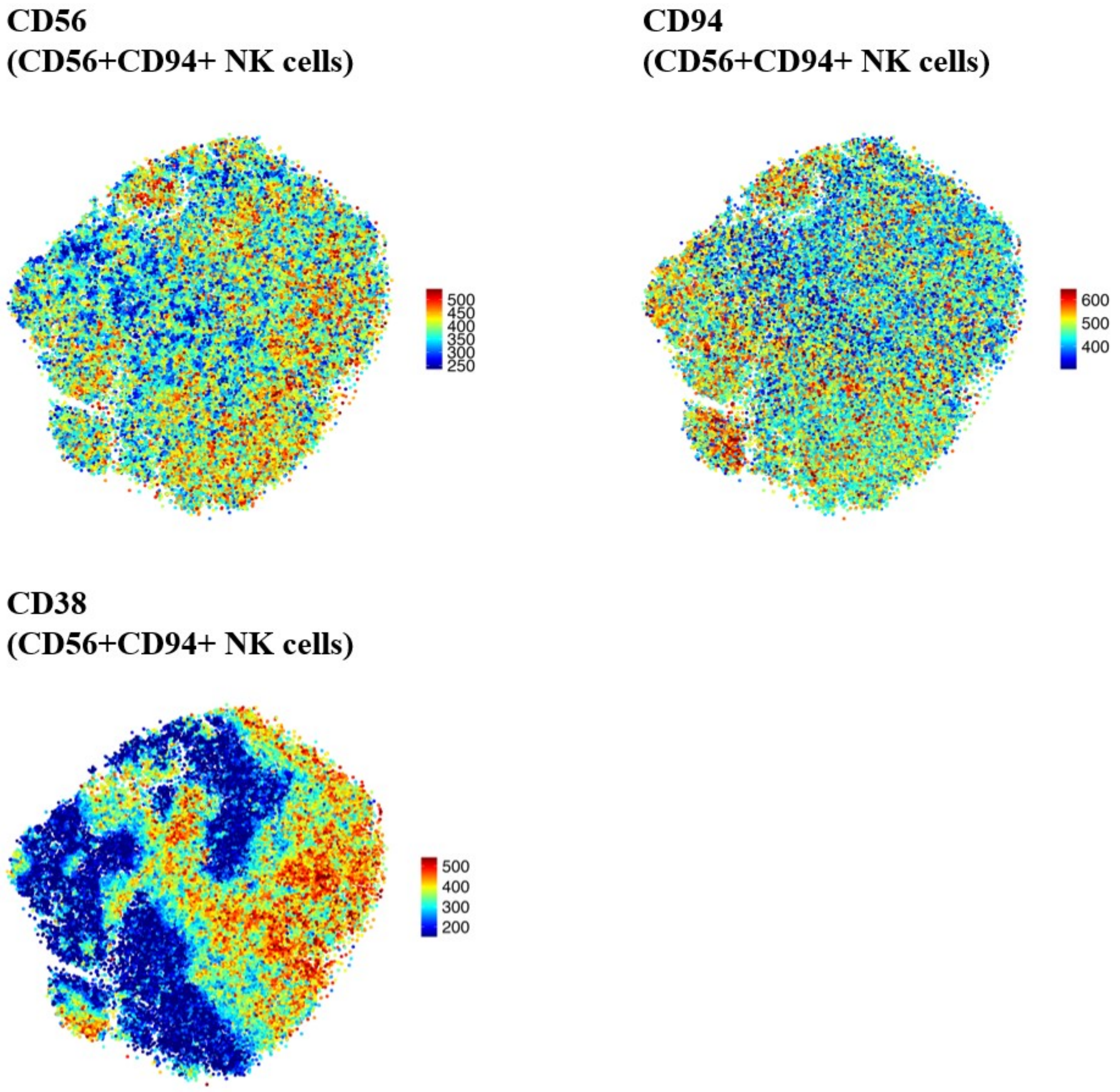

Supplementary Figure S6: CD38 expression does not correlate with CD56 or CD94 expression among CD56+CD94+ NK cells.

Expression levels of CD56, CD94 and CD38 among CD56+CD94+ NK cells on Fast Interpolation-based t-stochastic neighbor embedding (FIt-SNE) plots. A subsample of up to 70,000 cells from all study participants (non-MS controls, MS patients from all timepoints and batch controls) were included for the generation of FIt-SNE plots.

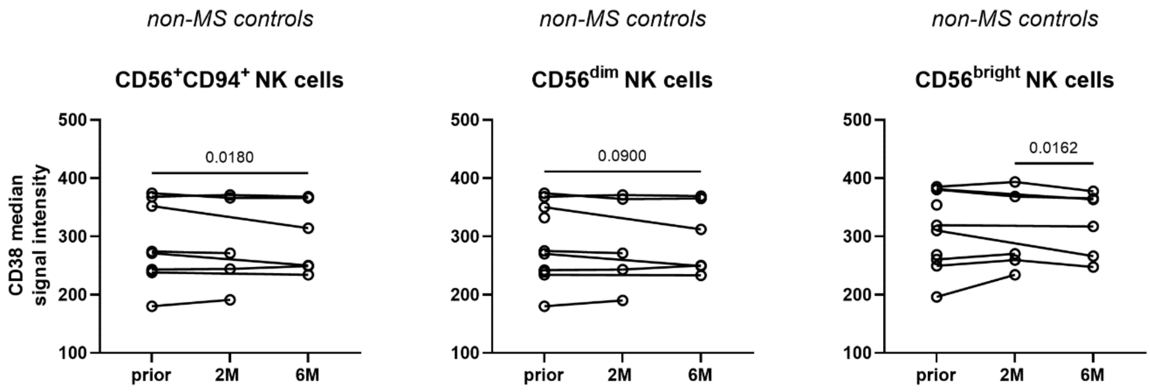

Supplementary Figure S7: CD38 median signal intensity among Lin-CD56+CD94+ NK cell subsets in non-MS control subjects.

Median signal intensity of CD38 among total CD56+CD94+ NK cells, as well as CD56dim and CD56bright NK cell subsets was calculated at baseline (*prior*, n = 10), and *2M* (n = 5) and *6M* (n = 6) from baseline in non-MS control subjects. A linear mixed-effects model was calculated for comparison of different study timepoints. 4,999 permutations were then run to calculate p-values. Holm's corrections were used for multiple comparisons which were performed to compare the different timepoints (*prior*, *2M* & *6M*). P-values of p<0.1 are indicated on the graphs.

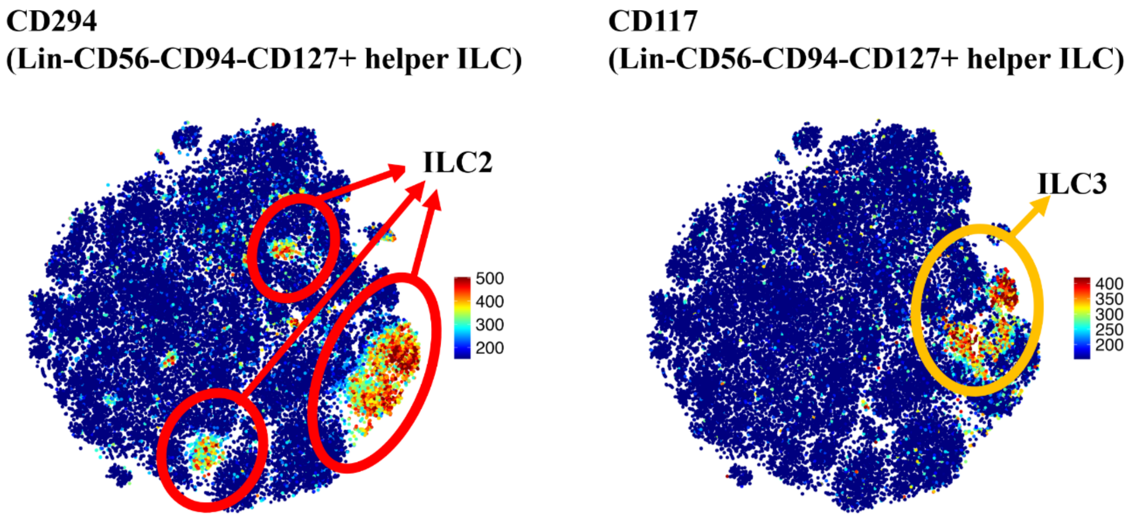

Supplementary Figure S8: Identification of Lin-CD56-CD94-CD127+ helper ILC subsets by FIt-SNE dimensionality reduction.

FIt-SNE plots generated on helper ILC (live CD45+ Lin- (CD3-CD19-CD14-CD11c-CD123-CD34-FcεRIa-TCRab-) CD127+CD56-CD94-). Expression levels of selected markers are shown, with helper ILC subsets ILC1 (CD294-CD117-), ILC2 (CD294+CD117+/-) and ILC3 (CD294-CD117+), as annotated.

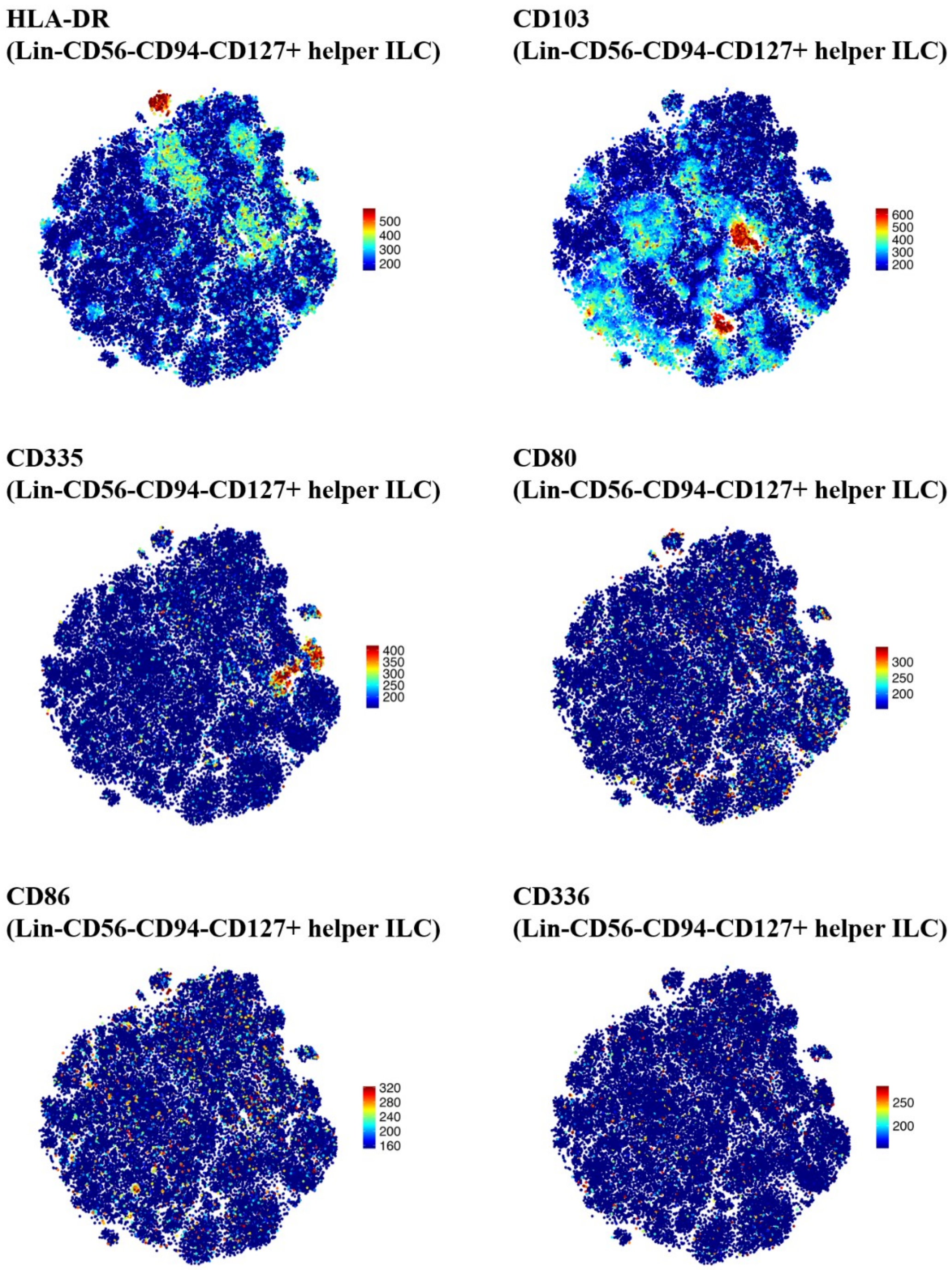

Supplementary Figure S9: Expression levels of various antibody panel markers among Lin-CD56-CD94-CD127+ helper ILC on FIt-SNE plots.

Expression levels of HLA-DR, CD103, CD335, CD80, CD86 and CD336 among Lin-CD56-CD94-CD127+ helper ILC on Fast Interpolation-based t-stochastic neighbor embedding (FIt-SNE) plots. A subsample of up to 70,000 cells from all study participants (non-MS controls, MS patients from all timepoints and batch controls) were included for the generation of FIt-SNE plots.

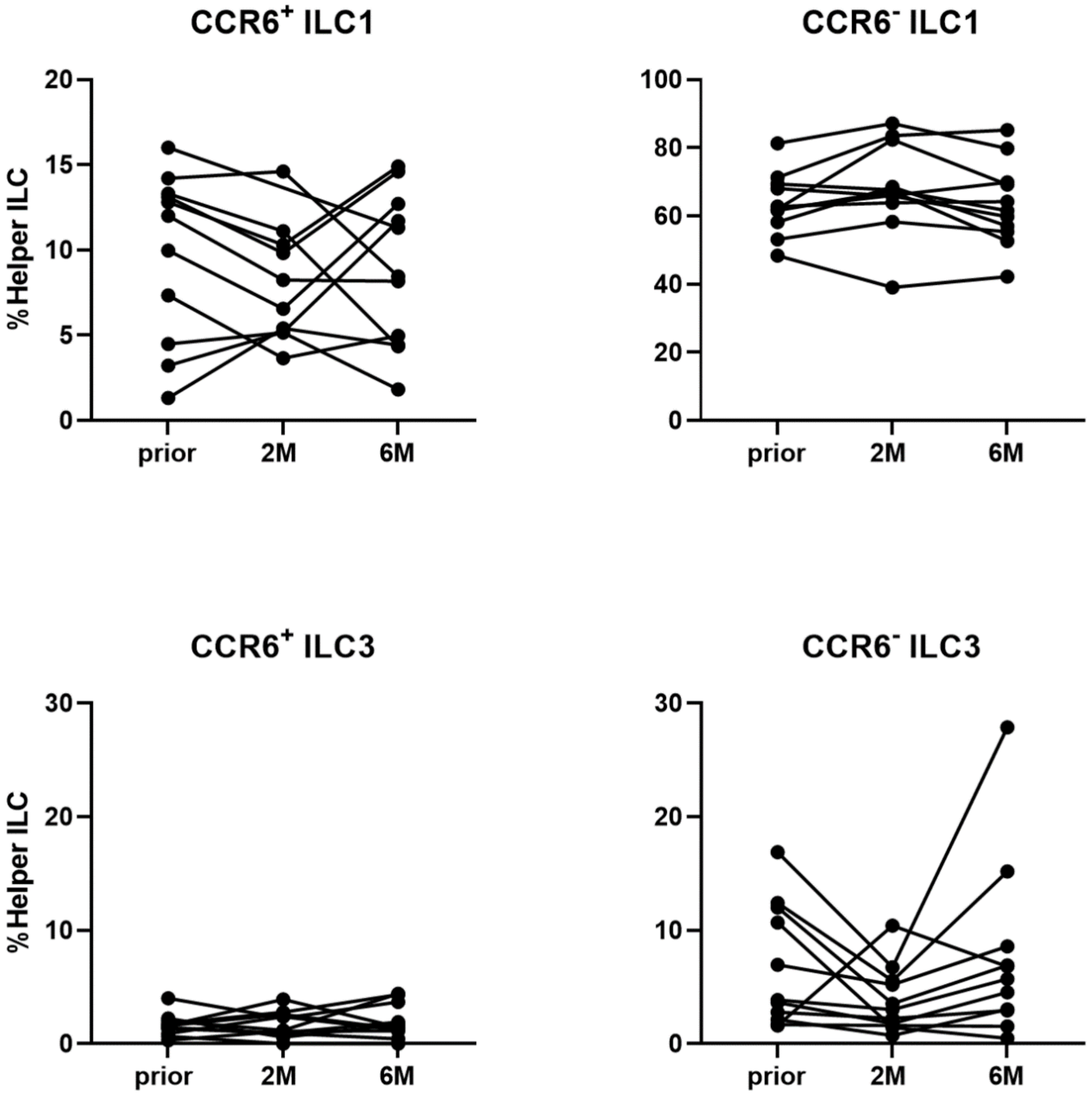

Supplementary Figure S10: Stable CD196+ (CCR6+) helper ILC subsets as proportion of Lin-CD56-CD94-CD127+ helper ILC following cladribine

CCR6+/- (CD196+/-) ILC1, and CCR6+/- ILC3 as %Lin-CD56-CD94-CD127+ helper ILC in MS patients at baseline (*prior*, n = 11), and two (*2M*, n = 10), and six months (*6M*, n = 11) following administration of cladribine. A linear mixed-effects model was calculated to compare between MS patients before and after treatment. 4,999 permutations were then run to calculate p-values. Holm's corrections were used for multiple comparisons which were performed to compare the different timepoints (*prior*, *2M* & *6M*). P-values of $p<0.1$ are indicated on the graphs.
